# Body composition, nutritional status, and endothelial function in physically active men without metabolic syndrome – a 25 year cohort study

**DOI:** 10.1186/s12944-016-0249-9

**Published:** 2016-04-27

**Authors:** Małgorzata Pigłowska, Tomasz Kostka, Wojciech Drygas, Anna Jegier, Joanna Leszczyńska, Mirosława Bill-Bielecka, Magdalena Kwaśniewska

**Affiliations:** Department of Geriatrics, Medical University of Lodz, Lodz, Poland; Department of Preventive Medicine, Medical University of Lodz, Zeligowskiego 7/9, 90-752 Lodz, Poland; Department of Epidemiology, Cardiovascular Disease Prevention and Health Promotion, Institute of Cardiology, Warsaw, Poland; Department of Sports Medicine, Medical University of Lodz, 92-213 Lodz, Poland

**Keywords:** Atherosclerosis, Body composition, Endothelial, Metabolic syndrome, Physical activity

## Abstract

**Background:**

The purpose of this analysis was to investigate the relationship between body composition, metabolic parameters and endothelial function among physically active healthy middle-aged and older men.

**Methods:**

Out of 101 asymptomatic men prospectively tracked for traditional cardiovascular risk factors (mean observation period 25.1 years), 55 metabolically healthy individuals who maintained stable leisure time physical activity (LTPA) level throughout the observation and agreed to participate in the body composition assessment were recruited (mean age 60.3 ± 9.9 years). Body composition and raw bioelectrical parameters were measured with bioelectrical impedance analysis (BIA). Microvascular endothelial function was evaluated by means of the reactive hyperemia index (RHI) using Endo-PAT2000 system.

**Results:**

Strong correlations were observed between lifetime physical activity (PA), aerobic fitness and most of analyzed body composition parameters. The strongest inverse correlation was found for fat mass (*p* < 0.01) while positive relationship for fat-free mass (*p* < 0.01), total body water (*p* < 0.05 for current aerobic capacity and *p* < 0.01 for historical PA), body cell mass (*p* < 0.001), muscle mass (*p* < 0.001), calcium and potassium (*p* < 0.01 and *p* < 0.001 for current aerobic capacity and *p* < 0.001 and *p* < 0.01 for historical PA, respectively) and glycogen mass (*p* < 0.001). Among metabolic parameters, HDL cholesterol (HDL-C) and uric acid were significantly associated with most body composition indicators. Regarding endothelial function, a negative correlation was found for RHI and body mass (*p* < 0.05) while positive relationship for RHI and body cell mass (*p* < 0.05), calcium (*p* < 0.05) and potassium mass (*p* < 0.05). Impaired endothelial function was observed among 8 subjects. Among bioelectrical parameters, impedance (Z) and resistance (R) normalized for subjects’ height were negatively related with body mass, body mass index (BMI) and waist circumference (*p* < 0.001); while reactance (Xc) normalized for patients’ height was negatively related with body mass (*p* < 0.05). The mean phase angle value was relatively high (8.83 ± 1.22) what reflects a good level of cellularity and cell function. Phase angle was positively related with body mass and BMI (*p* < 0.05).

**Conclusions:**

Both fat mass and muscle mass components are important predictors of metabolic profile. Maintaining regular high PA level and metabolically healthy status through young and middle adulthood may have beneficial influence on body composition parameters and may prevent age-related decrease of fat-free mass and endothelial dysfunction.

## Background

A large body of evidence shows that excessive weight and obesity are associated with cardiometabolic burden [1–3]. There are studies indicating that even comparable degree of overweight might be related with different cardiometabolic risk [4]. It seems that differences in body composition may better predict adverse cardiovascular diseases (CVD) events than simple weight-related indices [5].

Regular physical activity (PA) has been repeatedly shown as an independent factor in primary prevention of cardiometabolic disorders and atherosclerosis [6–9]. It has been documented that adequate physical level may beneficially modify major CVD risk factors including obesity and other metabolic disorders. The latest study of Laine et al. [10] performed among former elite athletes showed that vigorous leisure-time physical activity (LTPA) might protect from developing metabolic syndrome (MetS). Some other reports indicated that physically active lifestyle was among the most important factors explaining substantial cardiometabolic benefits of LTPA volume [9, 11].

However, most prior studies focused on simple anthropometric measurements which were operator-dependent and did not consider body composition in general [12]. There is a growing evidence that two major components of the body weight (i.e., fat mass and muscle mass) play an important role in predicting metabolic health. Body composition measurement extend the analysis of its impact on health outcomes independently of body mass index (BMI) categories [13, 14]. Recently, using raw electric data has gained popularity in nutrition assessment. Piccoli et al. [15] developed bioimpedance vector analysis (BIVA) method. The phase angle is the most specified impedance parameter for the diagnosis of malnutrition and clinical prognosis connected with changes in cellular membrane integrity as well as alterations in fluid balance [16]. Furthermore, the raw data may facilitate recognizing patients in different conditions [17–19].

Due to the prominent role of endothelial function in the development of early atherosclerosis, identifying individuals with endothelial dysfunction may improve risk stratification and prevent future CVD adverse events [20–23]. In our latest paper we found that even subtle changes in metabolic profile might influence microvascular endothelial function [24].

To our knowledge, no study has focused on the association between a large set of body composition variables, metabolic parameters and endothelial function in subjects with documented long-term regular LTPA level and metabolic profile. Prior studies that included more detailed assessment of body composition in relation to cardiometabolic risk, did not consider PA level of the participants [25, 26].

Therefore, the purpose of this analysis was to comprehensively investigate the relationship between body composition and cardiometabolic parameters as well as endothelial function among healthy physically active men. We hypothesized that higher fat mass, lower muscle mass and nutritional status would be associated with less beneficial metabolic profile and endothelial function.

## Methods

All the subjects were provided with a written information about the purpose and methodology of the study. The protocol of the project has been approved by the Medical University of Lodz Ethics Committee, and the written informed consent was obtained from all the participants. All clinical investigation have been conducted according to the principles expressed in the Declaration of Helsinki.

### Study design and subjects

Recruitment procedure and other methods were described in our latest paper [7]. The subjects of the study consisted of male volunteers who attended the Healthy Men Clinic and the Department of Preventive Medicine, Medical University of Lodz (Poland) from 1985, with the last regular examination taken in 2003. A total of 101 men (mean age 59.7 ± 9.0 years) met inclusion criteria for the 2012 follow-up structured check-up with comprehensive assessment of subclinical atherosclerosis. Subjects were considered to be eligible if before the examination they were asymptomatic, free from chronic diseases and treatment (including aspirin, statins and anti-hypertensive agents) and any important disability or dementia. As PA level may vary within individuals across time, we analyzed PA level throughout the whole observation. Based on the mean energy expenditure (EE) gathered at two-thirds of all follow-up examinations we defined subgroups of maintained, increased and decreased PA level. Out of 101 men participating in the whole project, 62 persons who maintained a stable LTPA level throughout the observation were invited to take part in the additional body composition assessment with bioelectrical impedance analysis (BIA). Finally, a total of 55 men agreed to participate in the BIA procedures (mean age 60.3 ± 9.9). The subjects were white men, predominantly married, white collar workers with university or secondary educational level whose occupational activity was low. Most participants had been involved in non-competitive sports activities of endurance type, such as running, bicycling, swimming, or basketball for several years.

### Protocol and measures

All subjects participated in a similar panel of procedures including a detailed interviewer-administered questionnaire, anthropometric and biochemical measurements, resting electrocardiogram and the graded submaximal exercise test. The 2012 follow-up included assessment of atherosclerosis indices and body composition analysis.

### Traditional cardiometabolic risk factors assessment

Fasting blood samples were drawn from the antecubital vein. Enzymatic methods were used to determine serum total cholesterol, glucose, triglycerides. HDL cholesterol (HDL-C) was measured by the precipitation method. Anthropometric data were collected by standard methods. Body mass was measured to the nearest 100 g on calibrated scales (in light indoor clothes and without shoes). Height was measured with a stadiometer (without shoes) to the nearest 0.5 cm. Waist circumference (WC) was measured with a tape measure at the middle of the distance between the lowest rib and the iliac crest (in underwear, standing position) to the nearest 0.5 cm. BMI was calculated as body mass (kilograms) divided by square of height (meters).

Metabolic disorders for males were defined according to the National Cholesterol Education Program Adult Treatment Panel III (NCEP ATP III) guidelines [27]. MetS was defined as the presence of 3 or more of the following parameters: WC ≥ 102 cm, systolic and/or diastolic blood pressure (BP) ≥ 130/85 mmHg, triglycerides (TG) ≥ 1.7 mmol/l, HDL-C < 1.0 mmol/l, fasting plasma glucose (FPG) ≥ 6.1 mmol/l.

### PA and aerobic capacity assessment

Data on PA was collected during the medical interview. The level of LTPA during the previous year was estimated. Exercise-related EE was calculated on the basis of the number of hours earmarked for weekly recreational sports activities (kcal/week) according to the tables of Fox et al. [28]. Historical PA was assessed according to Kriska [29] for the following periods of life: 12–34, 35–49, over 50 years old period, for the last 5 and 10 years and for the whole period from 12th year of life to the day of examination. All PAs were summed up according to hours per year, weeks of activity during the month, months of activity during the year, and years of activity during a period. The estimated number of hours during a period was divided by the number of years. As a result, all the measures of historical PA are expressed as hours per year for a given period.

In order to assess aerobic fitness the graded submaximal exercise test was carried out on a Monark type 818E (Stockholm, Sweden) bicycle ergometer with 30 W increments every 3 min to achieve at least 85 % of maximal age-predicted heart rate (220 - age). Heart rate (continuous ECG tracing) was regressed against the three last workloads. The resultant linear regression equation was used to calculate the aerobic capacity index, i.e. physical working capacity at 85 % of the maximal heart rate (PWC_85 % HRmax_). PWC_85 % HRmax_ was calculated by interpolating the workload-heart rate regression line at the point of 85 % of the maximal age-predicted heart rate. This methodology, even with lower (PWC_75 % HRmax_) exercise test intensity level, has been proposed as a useful measure of aerobic power for epidemiological studies [30]. PWC_85 % HRmax_ was expressed as relative to body mass [PWC/kg (W∙kg^−1^)].

### Body composition analysis

Multi-frequency bioelectrical impedance analysis (MF-BIA) was performed to assess body composition (BioScan 920-2, Maltron International Ltd). Whole body analysis was taken with subjects in a supine position in the right body side using eight surface electrodes, with the subjects having completed a minimum 6-h fast (in the morning, before breakfast) and according to other tips recommended by manufacturer. Data of sex, height, weight, age and race were entered into the device and the MF-BIA measurements were made. Saved data were downloaded using Maltron MF-BIA software and based on Maltron software’s equations the body composition of our subjects was assessed. Absolute values of the data obtained in BIA (fat mass, fat-free mass, total body water, body cell mass, muscle mass, protein mass, mineral mass, calcium mass, potassium mass, glycogen mass) were divided by body mass of the subjects and expressed in percentages of body mass. Resting metabolic rate (RMR) obtained in kcal was divided by subjects’ body mass and expressed in kcal/kg. Body volume was expressed in litres (L), body density in kg/L. We also present the ratio of extra/intracellular water and the nutritional index which is the ratio of extracellular mass and body cell mass. Additionally, the bioelectrical parameters of 50 kHz whole-body BIA: impedance (Z, Ohm), resistance (R, Ohm) and reactance (Xc, Ohm) normalized per subjects’ height (H) and the phase angle were analyzed. BIA measures whole-body Z which is the opposition of the body to alternating current. Z is a combination of R and Xc. R is inversely related to the volume of intra and extracellular ionic solutions, while Xc is directly related to soft tissue structures [15]. Phase angle is the arc tangent of Xc/R and therefore, it is dependent on pure resistive behavior of tissues (R) that mainly depends on tissue hydration and also on capacitive behavior of tissues (Xc) that is connected with cellularity, the size of cells, the cells’ membrane integrity [16, 31]. It reflects changes in the amount and the quality of soft tissue mass [16] and low phase angle indicates cell death or decreased cell integrity. The phase angle is positively associated with Xc and negatively associated with R [32].

### Endothelial function measurements

Peripheral arterial tonometry signals were obtained using the EndoPAT 2000 device (Itamar MedicalInc., Caesarea, Israel) in participants resting in the supine position in a quiet, temperature‐controlled environment set at about 22 °C after an overnight fast. Subjects were also instructed to refrain from smoking and strenuous exercise at least 12 h before the examination. Full details of the probe technology and the basis of measurements have been previously described [21]. Briefly, a PAT finger probe was placed on each index finger. Pulsatile volume changes of the distal digit induced pressure alterations in the finger cuff, which was sensed by pressure transducer and transmitted to and recorded by the EndoPAT 2000 device. Endothelial function was assessed via RH-PAT index. The ratio of the PAT signal after cuff release compared with baseline was calculated through a computer algorithm automatically normalizing for baseline signal and indexed to the contralateral arm. The estimated ratio reflects the RHI.

## Statistical analysis

Data was verified for normality of distribution and equality of variances. Spearman’s correlation was used to evaluate the association between LTPA characteristics, metabolic risk factors, RHI and body composition indices. The results of the quantitative variables are presented as mean ± SD (standard deviation). The paired t-test was used for comparison between continuous variables, and the χ 2 test or Fisher’s exact test was used for comparisons between categorical variables. The level of significance was set at *p* < 0.05 for all analyses.

## Results

Baseline characteristics and 25-year changes in traditional CVD risk factors and LTPA parameters are presented in Table 1. All the subjects remained generally metabolically healthy (with no or only one metabolic risk factors) throughout the observation. Most of the traditional CVD risk factors occurred less beneficial at follow-up, especially anthropometric characteristics, total and LDL-cholesterol (LDL-C) and FPG. However, HDL-C increased significantly during the observation (Table 1). None of the subjects developed MetS according to the ATP III definition during the whole observation. All subjects maintained their baseline PA during different life periods (12-34^th^, 35-49^th^, ≥50^th^, ≥12^th^ years of life and during last 5 and 10 years) as assessed by historical PA.

**Table 1:** 25-year changes in traditional cardiometabolic risk factors in the studied cohort

	Baseline	Follow-up
Age, years	35.3 ± 7.3	60.3 ± 9.9***
Waist circumference, cm	87.1 ± 7.8	95.4 ± 9.6***
BMI, kg/m^2^	24.7 ± 2.8	26.1 ± 3.2***
Systolic blood pressure, mmHg	122.9 ± 12.2	127.0 ± 13.2
Diastolic blood pressure, mmHg	78.6 ± 6.5	79.9 ± 7.04
Total cholesterol, mg/dl	186.0 ± 29.7	215.4 ± 36.3**
LDL-C, mg/dl	113.5 ± 22.8	132.9 ± 33.0**
Triglycerides, mg/dl	111.8 ± 37.2	101.1 ± 42.0
HDL-C mg/dl	50.1 ± 13.2	61.5 ± 19.1**
Fasting plasma glucose, mg/dl	82.8 ± 9.1	88.5 ± 8.7***
Uric acid, mg/dl	5.4 ± 1.2	5.9 ± 1.2
Metabolic syndrome, n	0	0
BP ≥ 130/85 mmHg, n	9	23**
Triglycerides ≥ 150 mg/dl, n	7	7
HDL-C < 40 mg/dl, n	3	3
Fasting plasma glucose ≥ 110 mg/dl, n	0	0
Current smokers, n	9	4
Exercise-related energy expenditure, kcal/week	4120.1 ± 3119	2979.2 ± 1826
PWC, W/kg	2.47 ± 0.5	2.0 ± 0.6*

Data presented as mean ± SD unless otherwise stated; * *p* < 0.05; ** *p* < 0.01; *** *p* < 0.001

*BMI* body mass index, *LDL-C* low-density lipoproteins, *HDL-C* high density lipoproteins, *PWC* physical working capacity

Table 2 shows body composition parameters and endothelial function indices at follow-up. The nutritional status of our sample is more favorable than generally observed in this age group. All of examined subjects were characterized with correct hydration status as well as extra- and intracellular water ratio and none of them was characterized with oedema. Impaired endothelial function (RHI < 1.67) was observed among 8 subjects.

**Table 2:** Body composition, bioelectrical impedance vector components and endothelial function in the studied cohort

	Follow-up
Fat mass (%)	24.8 ± 4.8
Fat-free mass (%)	75.2 ± 4.8
Total body water (%)	57.4 ± 4.1
Intracellular water (%)	57.0 ± 1.2
Body volume (L)	77.7 ± 12.0
Body density (kg/L)	1.0 ± 0.0
Resting metabolic rate (kcal/kg)	21.6 ± 2.2
Body cell mass (%)	41.7 ± 2.7
Muscle mass (%)	37.8 ± 4.4
Protein mass (%)	13.2 ± 1.8
Mineral mass (%)	4. 6 ± 0.6
Calcium mass (%)	1.6 ± 0.1
Potassium mass (%)	0.2 ± 0.0
Glycogen mass (%)	0.7 ± 0.1
Nutritional index	0.8 ± 0.0
Z, mean ± SD	444.9 ± 51.1
R, mean ± SD	440.1 ± 51.2
Xc, mean ± SD	67.9 ± 8.8
Phase Angle, mean ± SD	8.8 ± 1.2
Reactive hyperemia index, mean ± SD	2.0 ± 0.4
RHI <1.67, n	8

Abbreviations: *BMI* body mass index, *WHR* waist-to-hip ratio, *H* height, *R* resistance, *Xc* reactance, *Z* impedance

Both current and historical LTPA characteristics were significantly related to several analyzed body composition parameters assessed by BIA (Table 3). The strongest inverse correlation was found for fat mass while positive relationship for fat-free mass, total body water, body cell mass, muscle mass, total body potassium (TBK), calcium and glycogen mass. Aerobic power was substantially associated with several body composition characteristics. Higher PWC/kg was related to higher RMR, body cell mass, TBK, protein, mineral, calcium, glycogen mass as well as fat-free mass, body density, nutritional index and total body water. On the other hand, negative correlations were found between aerobic power and BMI, WC and body fat mass.

**Table 3:** Relationships between selected indicators of body composition and bioelectrical impedance vector components to PA characteristics, cardiometabolic parameters and endothelial function

	Current aerobic capacity	Historical PA	HDL-C	Systolic BP	Diastolic BP	Fasting plasma glucose	Uric acid	RHI
		≥12^th^ yr of life						
	W/kg							
Body mass (kg)	−0.147	−0.264*	−0.510***	0.078	0.317*	−0.028	0.373**	−0.287*
BMI (kg/m^2^)	−0.323*	−0.297*	−0.427**	0.206	0.344*	0.003	0.454***	−0.152
WC (cm)	−0.277*	−0.233*	−0.445**	0.122	0.314*	−0.018	0.296*	−0.182
Fat mass (%)	−0.449**	−0.368**	−0.339*	0.151	0.262	−0.027	0.374**	−0.188
Fat-free mass (%)	0.449**	0.368**	0.339*	−0.151	−0.262	0.027	−0.374**	0.188
Total body water (%)	0.287*	0.390**	0.372**	−0.050	−0.230	0.090	−0.340*	0.280
Extra/intracellular water (%)	0.191	0.002	0.135	−0.112	−0.141	−0.139	−0.297*	−0.086
Body volume (L)	−0.099	−0.214	−0.464***	0.043	0.257	0.009	0.330*	−0.229
Body density (kg/L)	0.449**	0.368**	0.339*	−0.151	−0.262	0.027	−0.374**	0.188
Resting metabolic rate (kcal/kg)	0.647***	0.345*	0.396**	−0.279*	−0.317*	−0.006	−0.306*	0.133
Body cell mass (%)	0.462***	0.486***	0.443**	−0.163	−0.336*	0.144	−0.438**	0.321*
Muscle mass (%)	0.510***	0.400**	0.337*	−0.215	−0.401**	0.081	−0.425**	0.198
Protein mass (%)	0.480***	0.090	0.096	−0.282*	−0.218	−0.034	−0.162	0.009
Mineral mass (%)	0.479***	0.093	0.095	−0.279*	−0.215	−0.032	−0.164	0.010
Calcium mass (%)	0.422**	0.466***	0.479***	−0.157	−0.355**	0.105	−0.471***	0.340*
Potassium mass (%)	0.453***	0.421**	0.400**	−0.185	−0.308*	0.102	−0.493***	0.345*
Glycogen mass (%)	0.551***	0.455***	0.402**	−0.240	−0.348*	0.095	−0.468***	0.064
Nutritional index	0.420**	0.052	0.050	−0.200	−0.120	−0.093	−0.169	−0.055
Z/H	−0.143	−0.016	0.308*	0.031	−0.149	−0.105	−0.153	0.034
R/H	−0.150	−0.047	0.307*	0.020	−0.157	−0.128	−0.171	0.011
Xc/H	−0.189	0.115	0.296*	−0.019	−0.080	0.033	−0.024	0.115
Phase Angle	−0.047	0.157	0.002	0.046	0.029	0.124	0.204	0.146

* *p* < 0.05 ** *p* < 0.01 *** *p* < 0.001

Abbreviations: *BMI* body mass index, *H* height, *R* resistance; *Xc* reactance, *Z* impedance

Among the studied metabolic risk factors, HDL-C and uric acid were substantially related to majority of the body composition parameters (Table 3). The strongest negative correlations were found between HDL-C and body mass, BMI, WC and body volume; and positive correlations for HDL-C and total body water, RMR, body cell mass as well as calcium, potassium and glycogen mass. According to the bioelectrical data, HDL-C was positively related with Z/H, R/H and Xc/H. Uric acid occurred positively related with body and fat mass while negatively fat-free mass, body density, body cell mass, muscle mass as well as calcium, potassium and glycogen mass.

Regarding endothelial function, a negative correlation was found for RHI and body mass while positive relationship for RHI and body cell mass, calcium and potassium mass (Table 3).

Both Z/H and R/H were strongly negatively related with body mass, BMI and waist circumference (*p* < 0.001). There was significant negative correlation between Xc/H and body mass (*p* < 0.05) while phase angle was positively related with body mass and BMI (*p* < 0.05) (data not shown in the Table).

## Discussion

In the present study we found significant relationship between LTPA volume (both current and historical) and majority of the analyzed parameters assessed by BIA. In our prior paper [7] we also showed strong association between BMI, WC and percentage of body fat and lifetime PA in all analyzed life periods. Similar findings were reached by Chrzczanowicz et al. [33] who showed that historical PA had a favorable effect on BMI, waist-to-hip ratio (WHR), WC and percentage of body fat. Strong positive correlation between LTPA and body cell mass supports previous findings on the protective role of PA in age-related decline in the actively metabolizing cellular components of the human body [34]. Higher level of LTPA was also associated with higher muscle mass and TBK content in a 3-year longitudinal study on body composition changes in the elderly men [35]. Our study cohort had comparable fat-free mass, body cell mass and TBK with healthy Caucasian men aged 35–59 years participating in the study by Kyle et al. [36].

Our study support previous findings indicating that differences in body composition and fat distribution might result in lower cardiometabolic risk among highly active individuals [4, 5]. According to Laine et al. [10], high volume of lifetime and current LTPA were associated with lower body fat level and risk for MetS among former male athletes as compared with their sedentary controls. In our metabolically healthy cohort, HDL-C, uric acid and BP (especially diastolic BP) occurred significantly related to most parameters assessed by BIA. Interestingly, we found potentially protective role of muscle mass in developing metabolic disorders which is consistent with the findings of Atlantis et al. [26].

Several studies demonstrated that BIVA approach might be an useful tool to assess nutritional status in various conditions, including heart failure, sarcopenia, or Alzheimer disease [18, 37–40]. Many available studies show negative correlations between bioelectrical parameters normalized to subjects’ height and BMI [41, 42]. Our results show that R/H and Z/H decrease with increasing BMI, but the correlation was not statistically significant for Xc/H. The phase angle is suggested to be an index of nutritional status [38], even better than anthropometric measurements or serum markers [43], which decreases with worsening of the nutritional status [16]. Cellular membrane stability may be also affected by intense physical training [44, 45]. Phase angle values in healthy people ranges between 5.0 and 7.0 [31] and values over 9.5 are possible to reach in some athletes [44]. The mean phase angle value of our sample was relatively high (8.83 ± 1.22) what reflects a good level of cellularity and cell function. It has also been suggested that a positive correlation between phase angle and BMI might be connected with increased number of muscle and fat cells [31]. In our population the phase angle was positively correlated with both body mass and BMI what is in agreement with the study performed by Torres et al. [44] in younger male elite athletes aged 13–48 years old and Micheli et al. [45]. We did not find the relationship between phase angle and LTPA what may be connected with the fact that all subjects in our study were characterized with high level of PA.

It is well documented that regular PA produce beneficial changes in lipids profile, including the influence on concentration and size of lipoprotein subclass [46]. During our over 25-year observation HDL-C concentration increased substantially as a result of long-term regular exercise trainings. As expected, HDL-C was significantly associated with majority of body composition parameters indicating that higher concentrations of HDL-C correlated with more beneficial BIA indices. Additionally, we observed a positive correlation between R/H, Xc/H, Z/H and HDL-C. The only study investigating the relationship between lipids and phase angle was performed by Dorna et al. in HCV-infected patients [47]. Their analysis showed no association between lipid profile and phase angle which is, to some extent, in line with our results.

In the available literature there are few studies investigating the relationship between body composition characteristics and endothelial function measured by RHI. Most previous studies focused on simple anthropometric indicators and used flow-mediated dilation (FMD) as a method reflecting vascular endothelium status. Results obtained by other authors indicate that the relationship between body fat and vascular function might become apparent when over fatness is evident. For example, significant inverse relationship between body fat and FMD among overweight and obese subjects [48–50] and correlation between BMI and endothelial dysfunction among obese adults with metabolic disorders (*p* < 0.001) [51] were observed. However, in the study of 50 healthy lean men aged 16–49 correlations between FMD and total, lean and fat mass were insignificant [52]. Relatively low prevalence of metabolic disorders in our study cohort may contribute to insignificant results within fat-related characteristics. Interestingly, we found positive correlation between RHI and some lean body parameters like body cell mass, TBK and total body calcium mass. Oberleithner et al. [53] in their study reported that an increase of extracellular potassium concentration significantly diminishes the stiffness of endothelial cells and has an influence on determining the physical compliance of endothelial cells. Furthermore, increases in potassium concentration to values such as those that occur during physical exercise in muscle greatly soften endothelial cells [54]. Body composition parameters presented in our analysis are more favorable than generally observed in this age group.

Several shortcomings of the present study should be acknowledged. The study is limited by its cross-sectional design which does not enable to draw definite causal relationships. Although the number of participants was relatively small, several substantial results were found. Well known limitation is related to self-reported questionnaires on PA which are prone to recall bias. The results obtained in this specific cohort may be representative only to healthy physically active middle-aged and older men. Of note, BIA method is not considered as a ‘gold standard’ for body composition assessment. However, it has been shown that BIA was a good predictor of DEXA-derived fat-free mass [55].

The distinct strength of the study is long-term observation of LTPA level and cardiometabolic risk factors. We are unaware of any studies investigating the relationship between metabolic risk factors, endothelial indices and such a wide set of body composition and nutritional parameters. Additionally, the BIA method was complemented with raw bioelectrical data that may be used for the monitoring the nutritional status. Precise selection of the participants as well as comprehensive assessment of healthy behaviours and clinical characteristics during the observation are important advantages of the analysis. Importantly, longitudinal observation of a homogenous group enables to eliminate the risk associated with such known confounders such as age, social class or lifestyle choices. We excluded individuals taking drugs modifying cardiometabolic risk in order to reduce the confounding effect of anti-atherogenic treatment.

## Conclusions

In summary, maintaining stable high LTPA and metabolically healthy profile through young and middle-adulthood are associated with beneficial body composition and nutritional status.

Maintaining high level of PA in middle aged and older men may have a favorable effect on cellular integrity and fluid balance what is expressed as a high phase angle that reflects the amount and the quality of soft tissue mass. Moreover, significant relationship between RHI and some lean body parameters may suggest that healthy lifestyle prevent age-related endothelial dysfunction and decrease of fat-free mass in the elderly.

## References

[1] Wilson PW, D’Agostino RB, Sullivan L, Parise H, Kannel WB (2002). Overweight and obesity as determinants of cardiovascular risk: the Framingham experience. Arch Intern Med.

[2] Su TT, Amiri M, Hairi FM, Thangiah N, Dahlui M, Majid HA (2015). Body composition indices and predicted cardiovascular disease risk profile among urban dwellers in Malaysia. BioMed Res Int.

[3] Shulman GI (2014). Ectopic fat in insulin resistance, dyslipidemia, and cardiometabolic disease. N Engl J Med.

[4] Laine MK, Eriksson JG, Kujala UM, Wasenius NS, Kaprio J, Bäckmand HM (2014). A former career as a male elite athlete-does it protect against type 2 diabetes in later life?. Diabetologia.

[5] Després JP (2012). Body fat distribution and risk of cardiovascular disease: an update. Circulation.

[6] Sofi F, Capalbo A, Cesari F, Abbate R, Gensini GF (2008). Physical activity during leisure time and primary prevention of coronary heart disease: an updated meta-analysis of cohort studies. Eur J Cardiovasc Prev Rehabil.

[7] Kwaśniewska M, Jegier A, Kostka T, Dziankowska-Zaborszczyk E, Rębowska E, Kozińska J (2014). Long-term effect of different physical activity levels on subclinical atherosclerosis in middle-aged men: a 25-year prospective study. PLoS One.

[8] Li J, Siegrist J (2012). Physical activity and risk of cardiovascular disease--a meta-analysis of prospective cohort studies. Int J Environ Res Public Health.

[9] Haskell WL, Lee IM, Pate RR, Powell KE, Blair SN, Franklin BA (2007). Physical activity and public health: updated recommendation for adults from the American College of Sports Medicine and the American Heart Association. Circulation.

[10] Laine MK, Eriksson JG, Kujala UM, Kaprio J, Loo BM, Sundvall J (2015). Former male elite athletes have better metabolic health in late life than their controls. Scand J Med Sci Sports.

[11] Samitz G, Egger M, Zwahlen M (2011). Domains of physical activity and all-cause mortality: systematic review and dose-response meta-analysis of cohort studies. Int J Epidemiol.

[12] Jung HS, Chang Y, Eun Yun K, Kim CW, Choi ES, Kwon MJ (2014). Impact of body mass index, metabolic health and weight change on incident diabetes in a Korean population. Obesity (Silver Spring).

[13] Lang PO, Trivalle C, Vogel T, Proust J, Papazian JP (2015). Markers of metabolic and cardiovascular health in adults: Comparative analysis of DEXA-based body composition components and BMI categories. J Cardiol.

[14] Lang PO, Trivalle C, Vogel T, Proust J, Papazyan JP, Dramé M (2015). Determination of cutoff values for DEXA-based body composition measurements for determining metabolic and cardiovascular health. Biores Open Access.

[15] Piccoli A, Rossi B, Pillon L, Bucciante G (1994). A new method for monitoring body fluid variation by bioimpedance analysis: the RXc graph. Kidney Int.

[16] Barbosa-Silva MCG, Barros AJD, Wang J, Heymsfield SB, Pierson RNJ (2005). Bioelectrical impedance analysis: population reference values for phase angle by age and sex. Am J Clin Nutr.

[17] Marini E, Buffa R, Saragat B, Coin A, Toffanello ED, Berton L (2012). The potential of classic and specific bioelectrical impedance vector analysis for the assessment of sarcopenia and sarcopenic obesity. Clin Interv Aging..

[18] Buffa R, Mereu RM, Putzu PF, Floris G, Marini E (2010). Bioelectrical impedance vector analysis detects low body cell mass and dehydration in patients with Alzheimer’s disease. J Nutr Health Aging.

[19] Toso S, Piccoli A, Gusella M, Menon D, Bononi A, Crepaldi G (2000). Altered tissue electric properties in lung cancer patients as detected by bioelectric impedance vector analysis. Nutrition..

[20] Simon A, Levenson J (2005). May subclinical arterial disease help to better detect and treat high-risk asymptomatic individuals?. J Hypertens.

[21] Rubinshtein R, Kuvin JT, Soffler M, Lennon RJ, Lavi S, Nelson RE (2010). Assessment of endothelial function by non-invasive peripheral arterial tonometry predicts late cardiovascular adverse events. Eur Heart J.

[22] Esper RJ, Nordaby RA, Vilariño JO, Paragano A, Cacharrón JL, Machado RA (2006). Endothelial dysfunction: a comprehensive appraisal. Cardiovasc Diabetol.

[23] Lerman A, Zeiher AM (2005). Endothelial function: cardiac events. Circulation..

[24] Kwaśniewska M, Kozińska J, Dziankowska-Zaborszczyk E, Kostka T, Jegier A, Rębowska E (2015). The impact of long-term changes in metabolic status on cardiovascular biomarkers and microvascular endothelial function in middle-aged men: a 25-year prospective study. Diabetol Metab Syndr..

[25] Wang J, Rennie KL, Gu W, Li H, Yu Z, Lin X (2009). Independent associations of body-size adjusted fat mass and fat-free mass with the metabolic syndrome in Chinese. Ann Hum Biol.

[26] Atlantis E, Martin SA, Haren MT, Taylor AW, Wittert GA, Members of the Florey Adelaide Male Ageing Study (2009). Inverse associations between muscle mass, strength, and the metabolic syndrome. Metabolism.

[27] Grundy SM, Cleeman JI, Merz CN, Brewer HBJ, Clark LT, Hunninghake DB, National Heart, Lung, and Blood Institute; American College of Cardiology Foundation; American Heart Association (2004). Implications of recent clinical trials for the National Cholesterol Education Program Adult Treatment Panel III guidelines. Circulation.

[28] Fox SM, Naughton JP, Gorman PA (1972). Physical activity and cardiovascular health. 3. The exercise prescription: frequency and type of activity. Mod Concepts Cardiovasc Dis.

[29] Kriska AM, Caspersen CJ (1997). Historical leisure activity questionnaire: a collection of physical activity questionnaires for health-related research. Med Sci Sports Exerc..

[30] Gore C, Booth ML, Bauman A, Owen N (1999). Utility of pwc75 % as an estimate of aerobic power in epidemiological and population-based studies. Med Sci Sports Exerc..

[31] Bosy-Westphal A, Danielzik S, Dörhöfer RP, Later W, Wiese S, Müller MJ (2006). Phase angle from bioelectrical impedance analysis: population reference values by age, sex, and body mass index. J Parenter Enteral Nutr.

[32] Baumgartner RN, Chumlea WC, Roche AF (1988). Bioelectric impedance phase angle and body composition. Am J Clin Nutr.

[33] Chrzczanowicz J, Gawron-Skarbek A, Kostka J, Nowak D, Drygas W, Jegier A (2012). Physical activity and total antioxidant capacity across an adult lifespan of men. Med Sci Sports Exerc.

[34] Dittmar M, Reber H, Hofmann G (2001). Age-related decline in body cell mass in elderly men and women, determined by a noninvasive nuclear technique: effects of physical activity and dietary potassium intake. Am J Hum Biol.

[35] Raguso CA, Kyle U, Kossovsky MP, Roynette C, Paoloni-Giacobino A, Hans D (2006). A 3-year longitudinal study on body composition changes in the elderly: role of physical exercise. Clin Nutr.

[36] Kyle UG, Genton L, Hans D, Karsegard L, Slosman DO, Pichard C (2001). Age-related differences in fat-free mass, skeletal muscle, body cell mass and fat mass between 18 and 94 years. Eur J Clin Nutr.

[37] Di Somma S, Lukaski HC, Codognotto M, Peacock WF, Fiorini F, Aspromonte N (2011). Consensus paper on the use of BIVA (Bioeletrical Impendance Vector Analysis) in medicine for the management of body hydration. Emerg Care J.

[38] Buffa R, Floris G, Marini E (2009). Bioelectrical impedance vector analysis in the assessment of nutritional status in the elderly. Nutr Ther Metabol.

[39] Norman K, Stobäus N, Pirlich M, Bosy-Westphal A (2012). Bioelectrical phase angle and impedance vector analysis--clinical relevance and applicability of impedance parameters. Clin Nutr.

[40] Pigłowska M, Gilbert T, Guligowska A, Ait S, Kostka T, Bonnefoy M (2015). Bioelectrical impedance vector analysis as an auxiliary method in diagnosing of sarcopenia among hospitalized older patients – a preliminary report. Eur Geriatr Med.

[41] Bosy-Westphal A, Danielzik S, Dörhöfer RP, Piccoli A, Müller MJ (2005). Patterns of bioelectrical impedance vector distribution by body mass index and age: implications for body-composition analysis. Am J Clin Nutr..

[42] Piccoli A, Pillon L, Dumler F (2002). Impedance vector distribution by sex, race, body mass index, and age in the United States: standard reference intervals as bivariate Z scores. Nutrition.

[43] Gupta D, Lammersfeld CA, Burrows JL, Dahlk SL, Vashi PG, Grutsch JF (2004). Bioelectrical impedance phase angle in clinical practice: implications for prognosis in advanced colorectal cancer. Am J Clin Nutr.

[44] Torres A, Oliveira K, Oliveira-Junior A, Gonçalves M, Koury J (2008). Biological determinants of phase angle among Brazilian elite athletes. Proc Nutr Soc.

[45] Micheli ML, Pagani L, Marella M, Gulisano M, Piccoli A, Angelini F (2014). Bioimpedance and impedance vector patterns as predictors of league level in male soccer players. Int J Sports Physiol Perform.

[46] Sarzynski MA, Burton J, Rankinen T, Blair SN, Church TS, Després JP (2015). The effects of exercise on the lipoprotein subclass profile: A meta-analysis of 10 interventions. Atherosclerosis.

[47] Dorna Mde S, Costa NA, Oliveira EP, Sassaki LY, Romeiro FG, Paiva SA (2013). Association between phase angle, anthropometric measurements, and lipid profile in HCV-infected patients. Clinics (Sao Paulo).

[48] Raitakari M, Ilvonen T, Ahotupa M, Lehtimäki T, Harmoinen A, Suominen P (2004). Weight reduction with very-low-caloric diet and endothelial function in overweight adults: role of plasma glucose. Arterioscler Thromb Vasc Biol.

[49] Sciacqua A, Candigliota M, Ceravolo R, Scozzafava A, Sinopoli F, Corsonello A (2003). Weight loss in combination with physical activity improves endothelial dysfunction in human obesity. Diabetes Care.

[50] Arkin JM, Alsdorf R, Bigornia S, Palmisano J, Beal R, Istfan N (2008). Relation of cumulative weight burden to vascular endothelial dysfunction in obesity. Am J Cardiol.

[51] Gupta AK, Ravussin E, Johannsen DL, Stull AJ, Cefalu WT, Johnson WD (2012). Endothelial dysfunction: an early cardiovascular risk marker in asymptomatic obese individuals with prediabetes. Br J Med Med Res.

[52] Hopkins ND, Green DJ, Tinken TM, Sutton L, McWhannell N, Cable NT (2010). Does brachial artery flow-mediated dilation scale to anthropometric characteristics?. Eur J Appl Physiol.

[53] Oberleithner H, Callies C, Kusche-Vihrog K, Schillers H, Shahin V, Riethmüller C (2009). Potassium softens vascular endothelium and increases nitric oxide release. Proc Natl Acad Sci U S A.

[54] Nordsborg N, Mohr M, Pedersen LD, Nielsen JJ, Langberg H, Bangsbo J (2003). Muscle interstitial potassium kinetics during intense exhaustive exercise: effect of previous arm exercise. Am J Physiol Regul Integr Comp Physiol.

[55] Roubenoff R (1996). Applications of bioelectrical impedance analysis for body composition to epidemiologic studies. Am J Clin Nutr.

